# A Review of SERS-Based Bacterial Detection from Nanomaterials to Integrated Clinical Platforms

**DOI:** 10.3390/bios16070396

**Published:** 2026-07-21

**Authors:** Yueqi Yang, Jing Li, Xinyi Hu, Zong Dai, Jianhe Guo

**Affiliations:** School of Biomedical Engineering, Shenzhen Campus, Sun Yat-sen University, Shenzhen 518107, China; yangyq98@mail2.sysu.edu.cn (Y.Y.); lijing329@mail2.sysu.edu.cn (J.L.); huxy97@mail2.sysu.edu.cn (X.H.)

**Keywords:** Surface-Enhanced Raman Scattering (SERS), bacterial detection, point-of-care testing, clinical diagnostics

## Abstract

Pathogenic bacterial infections remain a persistent global public health crisis. However, traditional clinical detection methods—such as culture-based assays and polymerase chain reaction (PCR)—are often time-consuming and labor-intensive, and they lack sufficient sensitivity for low-abundance pathogens, hindering rapid point-of-care diagnosis. With label-free, ultra-sensitive molecular fingerprinting, Surface-Enhanced Raman Scattering (SERS) has emerged as a powerful tool for rapid pathogen identification. This review summarizes the evolution of SERS-based bacterial detection from fundamental nanomaterials to integrated clinical diagnostic platforms. The article explores four core dimensions: functional integration and enrichment strategies of colloidal probes; structural design and multifaceted capture mechanisms of solid substrates; synergistic advantages of microfluidic systems in enabling automated “sample-to-answer” architectures; and the translational potential of SERS-based lateral flow assays (LFAs) for robust point-of-care testing (POCT). This review reveals a research shift from maximizing electromagnetic enhancement toward overcoming matrix effects in clinical samples and ensuring robustness. In synergy with microfluidics, LFAs, and AI, SERS technology is bridging the bench-to-bedside gap, offering a roadmap for next-generation decentralized, high-precision diagnostics.

## 1. Introduction

Surface-enhanced Raman scattering (SERS) has become a powerful analytical tool for biomedical applications [1,2,3]. By leveraging plasmonic nanostructures, SERS achieves extraordinary enhancement factors (10^6^ to 10^8^) [4], enabling the identification of analytes at the single-molecule level through their characteristic vibrational fingerprints [5,6]. Consequently, SERS is widely employed in drug discovery, food safety, and environmental monitoring.

Pathogenic bacterial infections remain a persistent global public health crisis, responsible for millions of infections and deaths each year [7,8]. To protect public health and assess treatment efficacy, rapid, accurate, and reliable identification of bacterial pathogens is essential [9]. However, traditional methods—including culture, polymerase chain reaction (PCR), and fluorescence bioassays—suffer from inherent limitations such as long turnaround times, complex sample preparation, and limited multiplexing capability [10,11], all of which impede timely clinical intervention [12].

To address these urgent challenges, SERS has emerged as a promising solution that effectively overcomes the shortcomings of conventional detection techniques [13]. SERS offers a cost-effective, rapid, and label-free approach for pathogen identification [14,15]. Its narrow spectral bandwidth and high molecular specificity provide “fingerprint” information that enables accurate bacterial differentiation [16,17,18] as well as simultaneous biomarker detection and single-cell analysis [19,20]. By integrating nanotechnology with Raman spectroscopy, SERS-active plasmonic nanostructures significantly improve detection limits [21,22,23]. Since Efrima and Bronk first reported the SERS spectra of *Escherichia coli* in 1998 [24], the technique has evolved into a sophisticated platform for on-site clinical diagnostics over recent decades.

Several recent reviews have summarized SERS-based pathogen detection from the perspectives of target species, nanomaterial design, sensing mechanisms, point-of-care (POC) formats, and chemometric or machine-learning approaches [25,26,27,28]. In contrast, the present review is structured around the workflow and platform evolution of SERS-based pathogen detection. Rather than treating colloidal probes, solid substrates, microfluidic devices [29], and lateral flow assay (LFA) strips [10] as discrete technical categories, we discuss how these platforms collectively and sequentially address the principal translational bottlenecks in clinical bacterial diagnostics—namely, signal generation, reproducibility, sample pretreatment, field deployability, and spectral interpretation. To articulate the conceptual distinction of this review, Table 1 compares representative SERS-related reviews with our work in terms of topical scope, organizational logic, and discussion focus. Moreover, the review concludes with a discussion and outlook on the integration of SERS with portable readers for POC diagnostics, the prospects of AI-assisted diagnostics and the translation of SERS bioassays from the laboratory to clinical settings.

## 2. Colloidal Probe-Based Bacterial Detection

### 2.1. Overview of Colloidal Carriers

**Figure 1:**
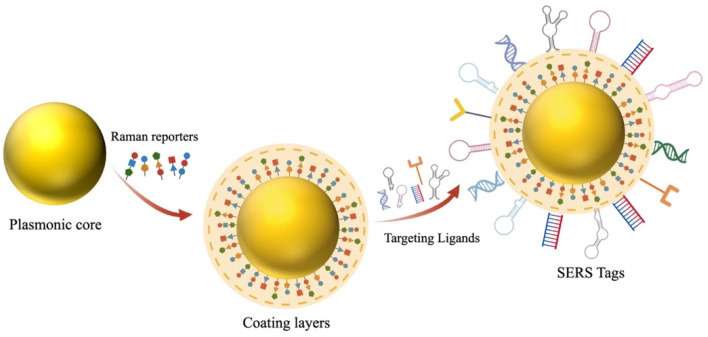
Building blocks and preparation process of a SERS tag.

In the early stage of Surface-Enhanced Raman Scattering-based (SERS-based) bacterial detection technological development, often referred to as the “pre-system” phase, colloidal carriers exist as free-floating nanoparticles (NPs) that serve as the primary sensing units. Their performance is governed by a tripartite architecture: (i) a plasmonic core composed of noble metals, which provides electromagnetic enhancement [30]; (ii) a Raman reporter or tag (e.g., 5,5′-dithiobis(2-nitrobenzoic acid) (DTNB), 4-mercantobenzoic acid (4-MBA)) that yields a distinct molecular fingerprint [31]; and (iii) a biorecognition layer (e.g., antibodies or aptamers) designed for specific pathogen capture [30]. As illustrated in Figure 1, a typical SERS tag is constructed by sequentially integrating a plasmonic core, Raman reporter molecules, a protective shell, and targeting ligands. The core amplifies the Raman signal, while the reporter molecules produce distinct spectral signatures; the shell not only improves particle stability but also provides conjugation sites, whereas the targeting ligands ensure specific pathogen binding.

Gold (Au) and silver (Ag) nanomaterials are widely used in SERS-based biosensing owing to their favorable optical properties [32,33]. Although Ag nanoparticles (NPs) provide stronger Raman enhancement, they are more prone to oxidation and instability than their Au counterparts. The sensing performance of these nanomaterials is largely governed by nanoparticle shape, which ranges from simple spheres to intricate star-like structures. Geometries with sharp features, such as nanostars [34] and spiky gold nanoshells [35], generate high-density “hotspots” that significantly boost sensitivity for bacterial strain identification. Despite these advances, balancing high sensitivity with batch-to-batch uniformity remains a challenge. Complex morphologies often compromise particle homogeneity and large-scale reproducibility. Consequently, recent research efforts have shifted toward the reproducible synthesis of nanoparticle structures with well-defined geometries to ensure consistent fabrication.

To enhance functionality, hybrid nanostructures are often developed; for example, the incorporation of magnetic components (e.g., Fe) enables efficient bacterial enrichment from complex sample matrices [36,37]. Combining noble metals with non-metallic materials has been demonstrated to further optimize enhancement and impart multifunctionality [38]. Recent breakthroughs have expanded the carrier library to include non-noble metal alternatives and advanced hybrid nanostructures. Platforms leveraging transition metal oxides (e.g., TiO_2_), semiconductors, sulfides, MXenes, and metal–organic frameworks (MOFs) have demonstrated increasing relevance for bacterial detection. These materials introduce complementary enhancement pathways, such as efficient photo-induced charge transfer (chemical mechanism) and high-density geometric enrichment, which minimize background fluorescence while providing extra physical or catalytic capabilities. For instance, heterostructures combining Ti_3_C_2_T_x_ MXene flakes with noble metal nanoparticles create interfacial junctions that facilitate strongly coupled chemical enhancement for the label-free fingerprinting of bacterial cells with high spectral reproducibility [39]. Concurrently, MOF-encapsulated plasmonic arrays act as porous scaffolds that serve as “molecular sieves” to selectively filter complex matrix interferences while capturing target bacterial cells or metabolites onto the active hot spots in a concentrated manner [40].

### 2.2. Functional Integration and Process-Oriented SERS Probe

Colloidal SERS-based bacterial detection has evolved from passive signal generation toward multifunctional, integrated diagnostic platforms. Modern probes now function as theranostic nanoplatforms, capable of orchestrating the entire workflow—from target recognition and enrichment to sensing and therapeutic pathogen inactivation. The integration of plasmonic metals with functional materials, such as magnetic components and aptamers, enables the detection of ultra-low levels of pathogens in complex matrices and facilitates rapid antimicrobial intervention.

#### 2.2.1. Integrated Targeting and Inactivation Platforms

Various SERS platforms have been designed to simultaneously enable SERS-based detection, target recognition, and in situ pathogen inactivation. The central premise of these strategies is to orchestrate multiple functionalities—plasmonic signal enhancement, photothermal conversion, and molecular specificity—within a single nanosystem. This synergistic integration directly addresses persistent challenges such as signal instability, matrix interference, and the conventional separation between diagnostics and therapeutics, thereby transitioning SERS probes from passive sensors toward active theranostic platforms that can perform the entire workflow from capture to quantification to intervention.

**Figure 2:**
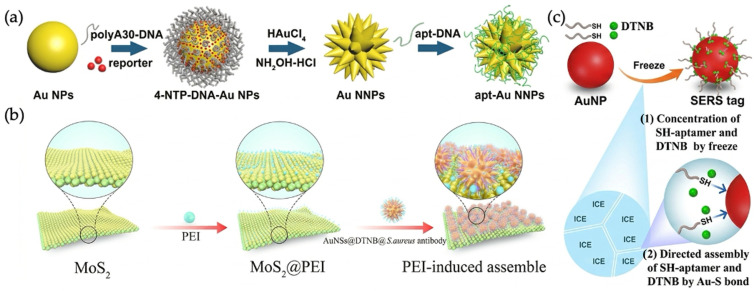
(**a**) The synthetic procedures of apt-Au NNPs. Reprinted with permission from ref. [41], Copyright 2026. (**b**) Illustration of the fabrication procedure of MoS_2_@AuNSs nanoflakes SERS tags. Reprinted with permission from ref. [42], Copyright 2026. (**c**) Construction of the aptamer/DTNB co-functionalized SERS tags through the co-freezing method. Reprinted with permission from ref. [36], Copyright 2026.

To integrate targeted recognition with immediate bactericidal activity, Huang et al. developed multifunctional Au nano-bridged nanogap particles (Au NNPs) in conjunction with Concanavalin A-modified magnetic nanoparticles for the specific capture of *Staphylococcus aureus* (Figure 2a) [41]. This robust affinity-based targeting, combined with carbohydrate-mediated magnetic isolation, effectively eliminates interference from the environmental matrix. Meanwhile, the core–shell nanogap architecture physically confines the Raman reporters within internal cavities, preventing reporter leakage and thereby ensuring stable baseline signals. The synergistic effect of biochemical recognition and structural design confers high analytical reproducibility, enabling complementary quantification via SERS and inductively coupled plasma mass spectrometry (ICP-MS) with a limit of detection (LOD) of 11 CFU/mL. Furthermore, the close proximity between the targeting probes and the bacterial surface allows for localized activation of the photothermal bactericidal component, thus bridging the gap between high-precision diagnostics and prompt therapeutic intervention.

Zhou et al. demonstrated a similar strategy that couples sub-cellular targeting with hyperthermia-driven pathogen eradication; they constructed multifunctional MoS_2_@Au nanostar (MoS_2_@Au NSs) nanoflakes engineered to target bacterial cell envelopes (Figure 2b) [42]. This localized surface affinity, combined with a ratiometric encoding strategy employing DTNB and 4-MBA as internal standards, effectively mitigates matrix interference, yielding a coefficient of variation (CV) of 1.57% and enabling detection at the single-cell level (1 cell/mL). Consequently, this precise on-cell localization ensures that the high photothermal conversion efficiency (43.41%) is directly delivered to the pathogen interface, resulting in 100% inactivation of free bacteria and 99% elimination of biofilms.

Given that the functional efficacy of these theranostic systems relies critically on the targeting domain, the rapid and oriented immobilization of biorecognition ligands on complex nanostructures remains a notable bottleneck. Xiao et al. addressed this challenge by introducing a simple and rapid co-freezing method for fabricating SERS signal probes (Figure 2c) [36]. Unlike conventional protocols that require several hours, this co-freezing approach exploits the voids created by ice microcrystals to assemble targeting aptamers and Raman reporters onto gold nanoparticles within just 8 min, conferring superior structural robustness and salt stability to the assembly. When combined with WGA-modified magnetic nanoparticles, this rapidly functionalized targeting architecture enables the rapid and sensitive detection of *Escherichia coli* O157:H7 and Mycobacterium tuberculosis DNA without compromising analytical performance.

#### 2.2.2. Efficient Enrichment and Pre-Concentration Strategies

The sensitivity of colloidal SERS probes is fundamentally constrained by the efficiency of effective collisions between the probe and the target pathogen. To enable the detection of rare bacteria in large clinical sample volumes, researchers have recently integrated magnetic responsiveness and topological trapping into multi-dimensional enrichment modules.

**Figure 3:**
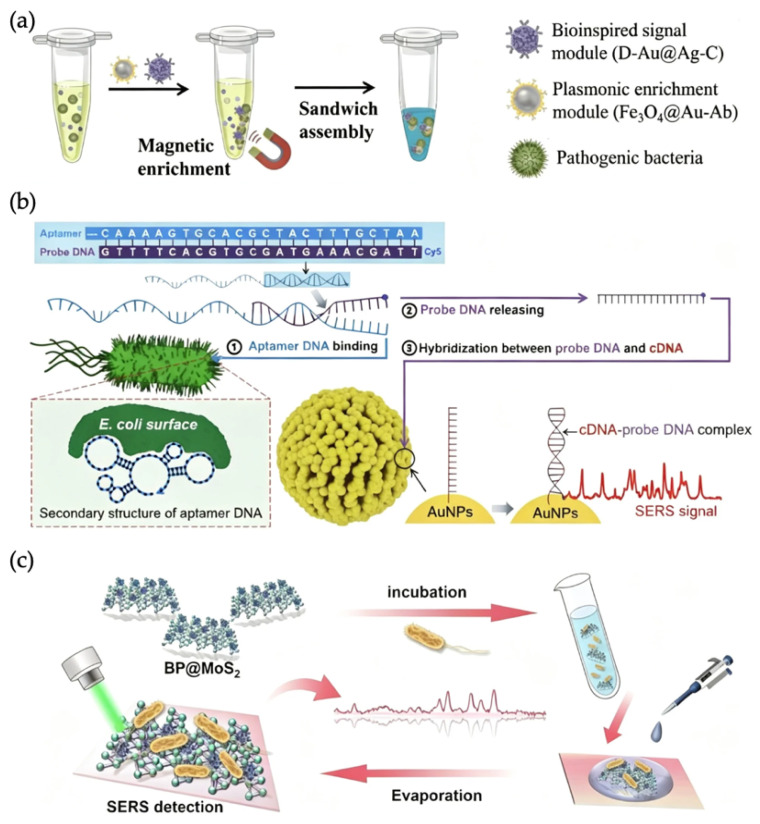
(**a**) Schematic illustration of bioinspired synergistic HS engineering strategy. Reprinted with permission from ref. [43], Copyright 2026. (**b**) Working principle of the SERS-based aptasensor. Reprinted with permission from ref. [44], Copyright 2026. (**c**) Schematic figure of label-free SERS detection and synergistic light-conversion depredation of pathogenic bacteria by BP@MoS_2_ nanocomposites. Reprinted with permission from ref. [45], Copyright 2026.

Wang et al. developed a bioinspired synergistic hot-spot engineering strategy to construct an ultrasensitive SERS sandwich bacterial sensor (USSB) [43] (Figure 3a). By loading plasmonic nanoparticles onto branched dendritic mesoporous silica nanoparticles (DMSNs), they optimized interparticle distances. This nanostructure, combined with a magnetic Fe_3_O_4_@Au enrichment module, maximized hot-spot density. This dual-module approach achieved an LOD of 7 CFU/mL for *Staphylococcus aureus* in mouse blood, thereby providing a robust tool for the early diagnosis of sepsis.

Moving from animal models to human clinical diagnostic efficacy, Kim et al. developed a culture-free SERS aptasensor specifically for the diagnosis of urinary tract infections (UTIs) [44]. To bypass the slow 48–72 h turnaround time of traditional culture methods, they used Au nanoparticle-embedded magnetic beads (MB-Au NPs) for bacterial enrichment and signal enhancement (Figure 3b). By employing a “signal-off to signal-on” mechanism involving the release of a labeled probe DNA upon bacterial binding, they circumvented sampling inconsistency caused by the size mismatch between bacteria and the Raman laser focal spot. Validated with 21 clinical samples, the assay achieved 100% sensitivity within just 6 h, demonstrating high clinical translational potential.

While the two studies described above rely on biological ligands, Liu et al. focused on the synergistic role of physical microstructures and semiconductor-based chemical enhancement [45]. Using a flower-like 2D BP@MoS_2_ hybrid (Figure 3c), they created high-surface-area “micro-traps” for label-free enrichment of *Escherichia coli.* Furthermore, this platform integrates a “detect-and-eliminate” functionality that harnesses the photothermal and photocatalytic properties of the 2D hybrid to achieve a sterilization rate of 99.66% within 8 min, thereby effectively bridging the gap between ultrasensitive label-free detection and on-site therapy.

#### 2.2.3. Specialized Detection: DNA, Phages and Metabolites

The integration of specialized biological components with signal amplification strategies has greatly enhanced the precision of colloidal SERS probes. Recent advances have shifted from simple surface-binding events toward dynamic DNA nanomachines, robust viral scaffolds, and interpretable metabolic profiling, enabling unprecedented sensitivity and molecular-level insights.

**Figure 4:**
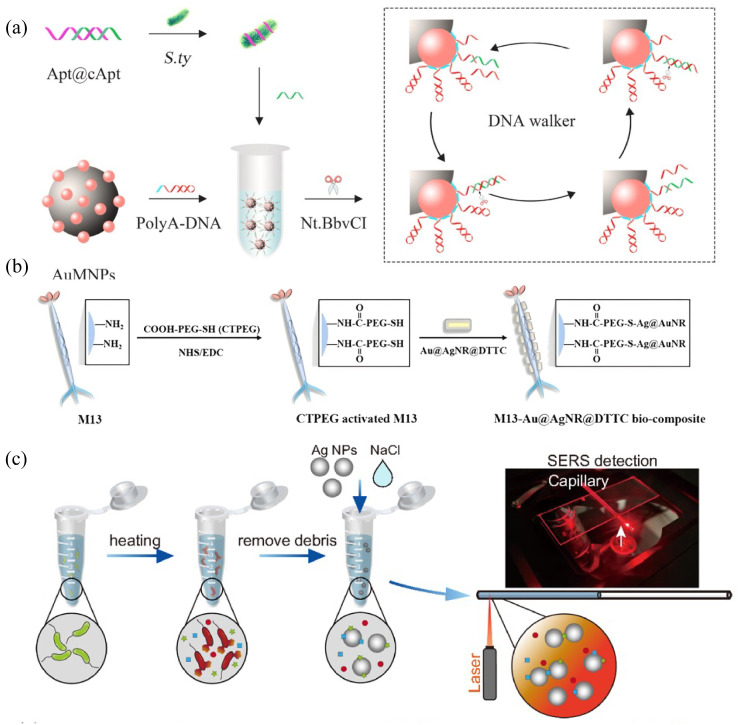
(**a**) Schematic illustration of the SERS strategy for bacterial detection. Reprinted with permission from ref. [46], Copyright 2026. (**b**) Fabrication of M13 phage-based SERS sensor. Reprinted with permission from ref. [37], Copyright 2026. (**c**) Schematic workflow including thermal lysis of bacteria, removal of debris, addition of Ag NPs and salt ions, injection of the mixture into a capillary tube, and detection using Raman spectroscopy. Reprinted with permission from ref. [47], Copyright 2026.

To address the low signal-to-noise ratio at trace analyte levels, Yang et al. developed a three-dimensional DNA walker on gold-modified magnetic nanoparticles (Au MNPs) [46] (Figure 4a). In the presence of Salmonella typhimurium, a target-triggered strand displacement reaction initiates cyclic motion mediated by an endonuclease. This “nanomachine” continuously cleaves DNA on the Au MNP surface, generating numerous binding sites for SERS tags. This novel signal amplification mechanism achieved an improved LOD of 4 CFU/mL. By isolating the signal from complex biological matrices, the platform ensures high reproducibility and minimizes background interference.

Although DNA nanostructures provide high sensitivity, their biological robustness remains a concern in harsh clinical settings. For signal-on/off assays based on target-triggered DNA displacement or nuclease-mediated DNA walkers, false-positive signals may arise from nonspecific strand release, nuclease contamination, DNA adsorption/desorption on nanoparticles, or endogenous biomolecules in clinical matrices. Therefore, matrix-matched negative controls, optimized washing or magnetic separation, nuclease-free conditions, and dual-recognition or ratiometric readout strategies are necessary to improve assay reliability in real samples.

As an alternative to DNA-based recognition and amplification, Bi et al. employed the M13 bacteriophage as a multifunctional biological scaffold [37] (Figure 4b). The authors leveraged the genetically modifiable pVIII capsid of the M13 phage to drive the self-assembly of Au@Ag core–shell nanorods (Au@Ag NRs) along its filamentous structure, creating a high density of electromagnetic hot spots. Simultaneously, the affinity of the pIII capsid for the *Escherichia coli* F-pilus ensured specificity. This scaffold achieved an LOD of 0.5 CFU/mL and a killing efficiency of 90%. The innovation lies in using the virus as a robust, inexpensive nanocarrier that functions without the need for antibodies or aptamers.

Moving toward label-free metabolic fingerprinting, Chen et al. (Figure 4c) addressed the “black box” of SERS by providing molecular-level spectral interpretation [47]. They introduced “SERSome”, an approach that integrates plasmonic colloids with a convolutional neural network (CNN) and laser desorption/ionization mass spectrometry (LDI-MS) to cross-validate SERS peaks against intracellular metabolites. This dual-modal method classified eight bacterial species with 90.44% accuracy. Their breakthrough lies in deconstructing the complex SERS spectrum into a “metabolite panel,” demonstrating that bacterial identification is driven by specific metabolic distinctions rather than by mere random surface vibrations.

### 2.3. Advantages and Limitations of Colloidal SERS Probes

Colloidal SERS probes offer distinct advantages for bacterial detection by operating in fluid environments. Unlike static solid substrates, free-floating nanoparticles dynamically interact with pathogens in three-dimensional volumes, significantly increasing collision rates in complex clinical matrices such as blood or urine. Engineered nanostructures, including nanostars and nanogaps, provide intense electromagnetic enhancement and single-cell sensitivity. Furthermore, their versatility enables the integration of multiple functions, including magnetic enrichment and photothermal therapy, thereby facilitating a closed-loop diagnostic and therapeutic system.

Nevertheless, several technical limitations hinder the universal clinical translation of these probes. Colloidal suspensions are inherently unstable; factors such as high salt concentrations or pH fluctuations in biological samples can trigger uncontrolled aggregation, leading to signal instability and poor reproducibility. Although Ag nanoparticles offer superior SERS enhancement, their susceptibility to oxidation compromises long-term reliability. Additionally, the absence of a rigid sensing interface results in random hot-spot distribution, complicating consistent quantification. These challenges underscore the need for rigorous fabrication protocols and hybrid materials that balance high sensitivity with the operational robustness required for point-of-care applications.

In addition, batch-to-batch uniformity remains a critical issue for clinical translation. Small variations in nanoparticle size, shape, surface ligand density, reporter loading, and aggregation state may lead to substantial differences in hot-spot distribution and SERS intensity. Therefore, standardized synthesis protocols, quality-control metrics, internal standards, and stability testing are necessary before colloidal SERS probes can be translated into clinical workflows. Closely related to this issue, SERS signal noise also affects quantitative reliability. Noise may originate from random hot-spot distribution, nanoparticle aggregation, fluorescence background, laser fluctuation, substrate heterogeneity, and matrix interference. Practical conventional noise-management strategies include internal standards, ratiometric readout, repeated spectral mapping, background subtraction, standardized spectral preprocessing, and matrix-matched controls. Various innovative approaches have also recently been developed to manage noise in SERS. Jeong et al. demonstrated that the spread spectrum SERS (ss-SERS) method encodes excitation light and decodes signals using peak autocorrelation, achieving over three orders of magnitude SNR improvement and enabling attomolar-level label-free neurotransmitter detection [48]. Meanwhile, in a separate study, Shin et al. demonstrated that rough-cutting the end surface of optical fibers significantly enhances SNR, with the roughened surface increasing SERS intensity while reducing background noise by approximately 32% compared to conventional flat-surface fibers [49]. More recently, Ranasinghe et al. introduced a noise-management-oriented design strategy using hybrid SERS substrates comprising Au nanoparticles and 2D materials [50]. They revealed that the surface conductivity and nanoparticle density distributions are the key parameters governing noise suppression. Collectively, these strategies offer complementary pathways for noise suppression in SERS-based sensing.

## 3. Solid Substrate-Based Bacterial Detection

### 3.1. Substrate Evolution and Design Principles

The development of Surface-Enhanced Raman Scattering (SERS) substrates has undergone a significant transition from random colloidal aggregates to highly ordered arrays and functionalized surfaces. Historically, from the 1970s to the 1990s, progress in the field was constrained by the difficulty of producing substrates that were both highly enhancing and reproducible [51]. Since the turn of the millennium, advances in nanofabrication have enabled the creation of more reliable platforms that meet the stringent requirements of medical bacterial detection [52].

**Figure 5:**
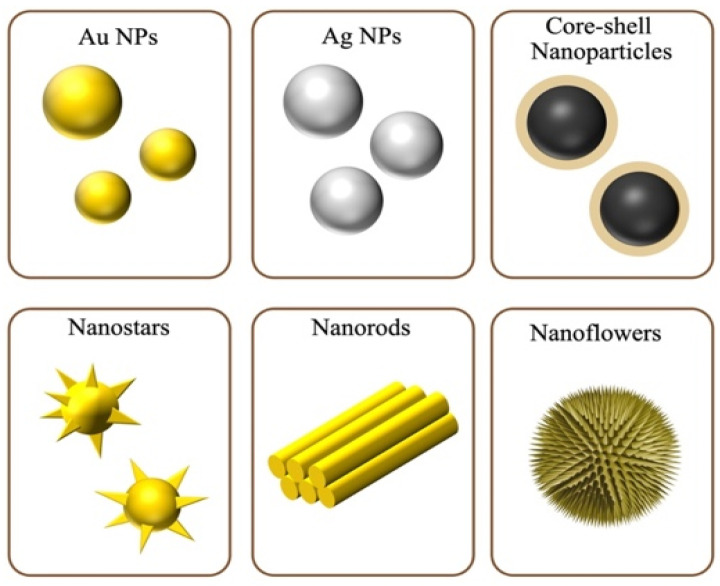
Diverse Nanoparticle Morphologies and Materials for SERS Detection.

The core design principle of solid substrates involves the precise control of morphology and material composition to generate high-density “hot spots” [29]. Researchers have explored a diverse library of materials, including single- and multi-component metals, semiconductors, two-dimensional materials, and complex composite nanostructures [53]. Such structural engineering allows for the fine-tuning of plasmonic properties. Figure 5 illustrates representative nanoparticles (NPs) of different materials and structures. Notably, substrate architecture significantly influences spectral signatures, underscoring the need for standardized design. By ensuring successful analyte binding, these customized substrates greatly enhance Raman signals, thereby enabling sensitive quantification and accurate structural identification in clinical samples [54].

### 3.2. Classification by Capture Mechanism

#### 3.2.1. Passive Capture

Passive capture involves directly applying a solution containing the target analytes onto SERS-active substrates and subsequently measuring their intrinsic SERS spectra. In this approach, detection relies solely on the characteristic SERS spectrum of the analyte, and the sensitivity and limit of detection (LOD) are determined by the enhancement factor (EF) of the SERS substrate [55]. This label-free strategy is highly valuable for clinical diagnostics, as it enables rapid identification via molecular fingerprinting without the need for sophisticated biochemical labeling.

**Figure 6:**
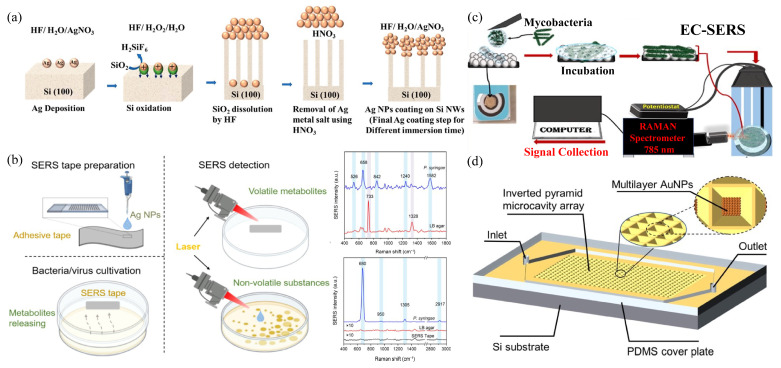
(**a**) Schematic diagram of the fabrication process of the utilized SERS substrate by means of the wet-chemical MACE technique. Reprinted with permission from ref. [56], Copyright 2026. (**b**) Schematic Illustration of SERS substrate preparation; Bacterial cultivation with/without virus and SERS detection of both volatile and nonvolatile metabolites. Reprinted with permission from ref. [57], Copyright 2026. (**c**) Method development for EC-SERS approach-1 (incbECSERS-1). Reprinted with permission from ref. [60], Copyright 2026. (**d**) Schematic diagram of the digital SERS chip. Reprinted with permission from ref. [61], Copyright 2026.

In the context of developing high-performance three-dimensional (3D) substrates, Das et al. fabricated a SERS chip comprising silver nanoparticles deposited on silicon nanowires (Si NWs) using metal-assisted chemical etching (MACE), aiming to overcome the limitations of conventional culture-based methods [56]. This high-aspect-ratio architecture (Figure 6a) provides a large surface area for bacterial attachment and hosts a dense 3D network of plasmonic hot spots. The platform achieved an exceptional LOD for bacteria as low as 100 CFU/mL, which is three orders of magnitude below the clinical threshold for urinary tract infections (UTIs). When integrated with a Siamese neural network, the system classified 12 bacterial species and distinguished antibiotic-resistant (AMR) strains in synthetic urine within 20 min, underscoring its potential for rapid clinical decision-making.

Crucially, passive capture strategies on solid substrates have recently transitioned from traditional liquid-phase bioassays to non-invasive gas-phase diagnostics by targeting bacterial volatile organic compounds (VOCs) and gaseous metabolites via headspace analysis. This emerging direction represents a culture-independent paradigm that circumvents complex liquid matrix interference and tedious sample pretreatment. For instance, Kelly et al. [57] developed a label-free headspace SERS method utilizing a specialized hydrophobic gold-nanoparticle-based substrate to passively capture indole (Figure 6b), a key volatile metabolic biomarker emitted by live pathogens. By monitoring the distinct vibrational fingerprints of indole gas diffusing onto the plasmonic surface, this platform achieved rapid discrimination between live Pseudomonas aeruginosa and *Escherichia coli* within only a few hours of culturing. Further expanding this gas-sensing capability for clinical monitoring, subsequent research [58] successfully tracked dynamic gas-phase bacterial metabolites to continuously profile microbial growth kinetics and establish distinct diagnostic signatures capable of differentiating bacterial respiratory infections from viral conditions.

To overcome the diffusion and sensitivity bottlenecks of trace gas analysis, Li et al. [59] recently designed and engineered an Au/TiO_2_-based SERS substrate possessing unique interface-driven hydrophobic interactions. This unique architecture facilitated the rapid, spontaneous, and ultrasensitive enrichment of highly volatile metabolic compounds from the headspace directly onto the active plasmonic junctions. This interface-mediated passive trapping strategy yielded exceptional spectral reproducibility and low detection limits, underscoring the immense clinical potential of gas-phase solid SERS frameworks for real-time, non-invasive pathogen bedside screening.

#### 3.2.2. Physical Field-Assisted Enrichment

Passive capture relies on natural sedimentation, whereas physical capture strategies employ active manipulation—such as microfluidic discretization, electrostatic, magnetic, or electrochemical forces—to actively transport and concentrate bacterial cells onto SERS-active surfaces. These strategies overcome diffusion limits and shorten assay times in complex or dilute clinical samples by functionalizing substrates or using external hardware for active pathogen “grasping.”

Electrochemical (EC) fields represent a key advancement for enhancing pathogen–surface affinity. As shown in Figure 6c, Hendricks-Leukes et al. proposed a dual-approach EC-SERS method for label-free identification of Mycobacterium tuberculosis (MTB) [60]. The innovation lies in a tailored, sequence-based voltage stepping protocol (positive-to-negative) that works in concert with polyelectrolyte-wrapped silver nanoparticles to mimic a natural conditioning layer. Unlike classical EC-SERS, this method uses applied potentials to manipulate local electric fields and promote irreversible adsorption of MTB’s waxy cell wall. This approach achieved discriminative detection of hypervirulent TB in unprocessed sputum and urine, marking a significant step toward decentralized pathogen identification.

Beyond external electrochemical fields, physical discretization through air displacement has proven to be an efficient strategy for active bacterial enrichment. Figure 6d illustrates a digital SERS chip that employs inverted pyramid microcavity arrays for rapid and accurate detection [61]. This approach partitions bacterial suspensions into thousands of picoliter (pL) cavities, concentrating bacteria within confined volumes to maximize metabolic signal accumulation. When combined with a multilayer gold nanoparticle (Au NP) substrate (enhancement factor ≈ 1.1 × 10^8^), the platform achieves precise label-free quantification of *Escherichia coli* within 4 h. By employing Poisson-based digital counting, this strategy mitigates analog signal fluctuations, thereby ensuring highly reliable and reproducible bacterial quantification on solid substrates.

Despite the current success of these electrical and magnetic strategies, optical field trapping (or optoplasmonic tweezers) represents a promising future direction for physical capture. Periodic nanostructures generate intense electromagnetic gradients, enabling laser radiation pressure to “lock” individual bacteria into hot spots. Although complex instrumentation currently limits clinical adoption, the non-contact, label-free manipulation of single cells offers transformative potential for studying pathogen heterogeneity and real-time drug responses.

#### 3.2.3. Chemical Affinity-Based Capture

Although physical field-based capture enables rapid pathogen manipulation, these techniques often lack molecular-level selectivity. To address this limitation, chemical capture strategies involve functionalizing solid substrates with specific ligands—such as boronic acids, antibiotics, or DNA sequences—to achieve analyte enrichment and precise localization within SERS hot spots.

**Figure 7:**
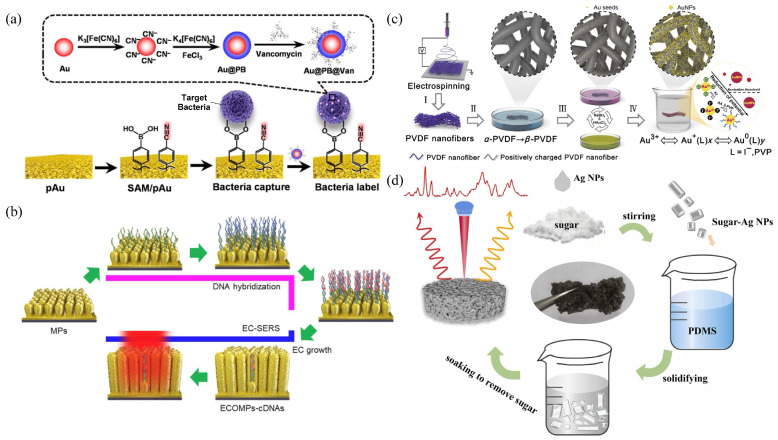
(**a**) Illustration of the Working Principle of the Present Multifunctional SERS Platform. Reprinted with permission from ref. [62], Copyright 2026. (**b**) Strategic diagram of bacterial detection based on the DNA hybridization-associated EC-SERS analytical method. Reprinted with permission from ref. [63], Copyright 2026. (**c**) Illustration of the fabrication process of the fiber membrane of PVDF@Au nanofiber in this work. Reprinted with the permission from ref. [64], Copyright 2026. (**d**) Schematic diagram for the preparation process of Ag NPs@PDMS sponge substrate. Reprinted with permission from ref. [65], Copyright 2026.

To advance clinical pathogen detection, Gao et al. developed an integrated SERS platform for the detection and removal of bacteria in whole blood [62]. The system employs a vancomycin-modified sandwich architecture targeting Gram-positive peptidoglycan (Figure 7a). In this design, the substrate is decorated with 4-mercaptophenylboronic acid (4-MPBA) to act as a chemical catcher for capturing bacteria. Crucially, Prussian Blue (PB) embedded within the Au@PB@Van SERS tags not only provides a distinguishable SERS band in the Raman-silent region to indicate bacteria levels, but also serves as a powerful photothermal agent. To overcome reproducibility issues arising from random hot spots, 4-mercaptobenzonitrile was used as an internal reference for ratiometric detection in the Raman-silent region, effectively shielding the signal from background noise originating in blood and proteins. This platform achieved high sensitivity for *Staphylococcus aureus* in blood and demonstrated theranostic potential by leveraging synergistic photothermal effects to eliminate trapped bacteria.

In addition to whole-cell capture, chemical strategies now target genetic markers that often fail to diffuse into narrow SERS hot spots. To address this challenge, Lee et al. developed an EC-SERS approach to engineer organometallic hot spots on nanopillars for the detection of bacterium-derived DNA [63] (Figure 7b). By hybridizing target sequences with thiol-terminated capture DNA on the nanopillars, they applied a redox potential to trigger the in situ deposition of gold ions around the DNA strands. This process traps the target molecules within dynamically generated hot spots, yielding a 2000-fold enhancement compared to passive substrates and achieving an LOD of 0.035 nM. This enabled successful discrimination of bacterial DNA in human whole blood.

#### 3.2.4. Structure-Assisted Capture

While physical and chemical capture strategies provide selectivity, the physical architecture of the substrate can be engineered to mechanically enhance pathogen retention and signal acquisition. This “structure-assisted capture” approach utilizes three-dimensional porous or fibrous frameworks to trap microorganisms, thereby maximizing the contact area between analytes and plasmonic hot spots.

Recognizing that conventional flat SERS surfaces often fail to excite the entire three-dimensional volume of micron-sized pathogens, a structural capture strategy employing electrospun flexible multilayered polyvinylidene fluoride (PVDF) nanofibers has been developed [64] (Figure 7c). Compact Au or Ag nanoparticles grown in situ on these high-curvature fibers create dense three-dimensional hot spots throughout the membrane. This 3D mesh physically encloses bacteria, ensuring direct localized surface plasmon resonance (LSPR) contact with the cell wall and overcoming the shadowing effects common in two-dimensional substrates. With an LOD of 0.1 nM for thiram and robust SERS signals from various bacteria, this flexible membrane represents a promising candidate for wearable or implantable biosensing.

Similar to fibrous membranes, hierarchical porous frameworks can effectively enrich analytes. Wang et al. developed a three-dimensional porous Ag NPs@PDMS sponge for label-free pathogen detection [65] (Figure 7d). Fabricated via a “mold transfer-surface embedding” method, the sponge’s internal pores provide a high surface area to trap microorganisms while protecting the Ag nanoparticles from oxidation. This design facilitates natural analyte accumulation, achieving an LOD of 1 CFU/mL for *Escherichia coli*. When combined with principal component analysis-support vector classification (PCA-SVC) machine learning, the system identified six microorganisms with 92.17% accuracy, demonstrating its potential for automated monitoring of waterborne infections.

### 3.3. Advantages and Limitations SERS Substrates

Solid substrates represent a strategic advancement in SERS-based diagnostics by offering high reproducibility and signal stability, as their fixed nanostructures eliminate the random aggregation and “signal hopping” typical of colloidal carriers. Although large-area fabrication may introduce minor structural non-uniformities arising from nano-templating defects, such spatial variations are substantially less severe and more readily manageable than the stochastic Brownian aggregation characteristic of dispersed colloidal suspensions. However, their low capture efficiency remains a significant limitation. The probability of a micron-sized bacterium in a three-dimensional volume effectively interacting with a two-dimensional sensing interface is governed primarily by diffusion-limited kinetics, which often necessitates prolonged incubation times or external driving forces.

Table 2 provides a simplified comparison of different SERS platforms. An inherent trade-off exists between capture specificity and universality. Although chemical affinity-based capture offers high selectivity, its clinical application is frequently hindered by high ligand costs and chemical instability. Conversely, physical enrichment strategies are more cost-effective but are more susceptible to non-specific capture of biological debris, which can contaminate the spectral background. Ultimately, the most daunting challenge for solid SERS platforms is the transition from laboratory-scale precision to industrial manufacturing. Achieving a high-density, even distribution of hot spots over large areas remains the primary obstacle to ensuring batch-to-batch reliability for clinical point-of-care applications.

## 4. Microfluidic Chip-Based Bacterial Detection

### 4.1. System Integration

The transition from static substrates to dynamic microfluidic “micro-lab-on-a-chip” systems has revolutionized bacterial diagnostics. In this context, microfluidics serves as a functional interface that enables the handling of complex clinical matrices, rather than merely acting as a passive carrier for plasmonic materials. These platforms streamline essential pre-analytical workflows—such as extraction, filtration, and preconcentration—by integrating Surface-Enhanced Raman Scattering active (SERS-active) matrices. This integration can be achieved either by injecting colloidal suspensions or by immobilizing solid substrates in designated areas, thereby enabling rapid detection with or without traditional culture methods [66,71].

The combination of SERS with channel-based chips or microfluidic paper-based analytical devices (µPADs) ensures successful system integration. This dynamic approach overcomes challenges related to signal reproducibility: by precisely manipulating fluid dynamics, these systems control particle aggregation and optimize the formation of electromagnetic “hot spots.” Such spatiotemporal control yields highly reproducible SERS responses that are unattainable in bulk colloidal assays [71].

The primary clinical advantage of this architecture is high-sensitivity analysis using minimal sample volumes [72], which is particularly valuable for point-of-care testing (POCT) applications. Recent research highlights that these integrated flow-SERS systems enable real-time pathogen identification [73]. Beyond detection, the synergy between microfluidics and SERS supports precise species discrimination, providing a robust, culture-independent tool for early clinical intervention [74].

### 4.2. Advanced Architectures for “Sample-to-Answer”

#### 4.2.1. On-Chip Pretreatment

**Figure 8:**
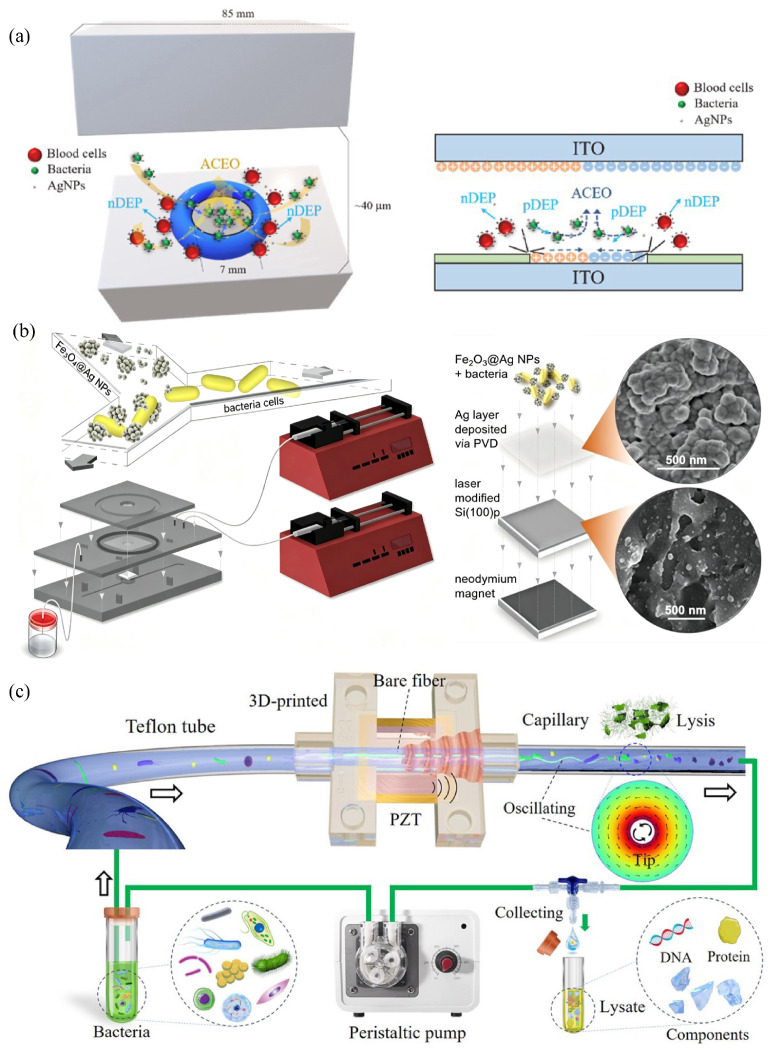
(**a**) The conceptual demonstration of the electrokinetic mechanism of selective concentration of bacteria in a single collector is shown in an overall view and a cross-section view. Reprinted with permission from ref. [75], Copyright 2026. (**b**) Mixing both types of solutions in a microfluidic chip and the adsorption of bacterial cells to Fe_2_O_3_@Ag NPs and formation of bacteria–NP aggregates. Reprinted with permission from ref. [76], Copyright 2026. (**c**) The acoustofluidic system is designed for rapid lysis of bacteria/cells. Reprinted with permission from ref. [77], Copyright 2026.

The incorporation of on-chip pretreatment modules into microfluidic SERS platforms represents a significant advance in direct clinical diagnostics, as it eliminates the labor-intensive centrifugation and time-consuming culture enrichment steps. As shown in Figure 8a, electrokinetic mechanisms enable selective bacterial concentration. Chen and Tseng’s 3D-ACEK/SERS system utilizes AC-electroosmosis (AC-EO) and dielectrophoresis (DEP) to identify bacteria directly from untreated whole blood [75]. This frequency-gated filter repels blood cells (10^8^ cells/mL) while focusing pathogens, achieving a 1000-fold concentration in 2 min. This yields a 3 CFU/mL limit of detection (LOD), significantly shortening the bacteremia diagnostic window. However, performance remains tied to the AC frequency response, requiring precise calibration for varying clinical sample conductivities.

Similarly, to address oral biofilm complexity, Witkowska and Kamińska’s group engineered a magnetomicrofluidic sensor to isolate pathogens from human saliva [76]. The researchers utilized bifunctional silver-coated magnetic nanoparticles (Fe_2_O_3_@Ag NPs) that first adsorb onto target bacterial surfaces for on-chip magnetic separation and subsequently serve as SERS enhancers by forming a “basket-like” hotspot structure when attracted to a Si/Ag platform (Figure 8b). This strategy avoids spectral overlaps in complex matrices, enabling species-level discrimination (82–91% accuracy) and a 10^3^ CFU/mL LOD within ~45 s without culturing. While rapid and selective, replacing external neodymium magnets with automated electromagnetic integration could further improve portability for point-of-care applications.

Beyond bacterial separation and enrichment, on-chip pretreatment can be extended to cell lysis for exposing intracellular biomolecules, a strategy highly compatible with both solid substrates and colloidal suspensions. Liu et al. addressed this limitation by developing an acoustofluidic lysis-enhanced SERS platform [77]. Their on-chip approach employs a torsionally oscillating fiber tip to generate high-intensity vortices within a capillary (Figure 8c). By concentrating bacteria in high-shear regions, this mechanism induces mechanical rupture of the bacterial membrane, thereby successfully exposing intracellular components—such as proteins, lipids, and nucleic acids—to the plasmonic surface. This pre-processing step improves spectral resolution compared to conventional whole-cell measurements. When coupled with a one-dimensional residual neural network (1D ResNet), the system achieved 98.9% classification accuracy across seven biological sample types using clinical throat swabs without the need for prior isolation, thereby enabling rapid, culture-free pathogen identification in real-world scenarios.

#### 4.2.2. Dynamic Target Capture and On-Site Concentration

**Figure 9:**
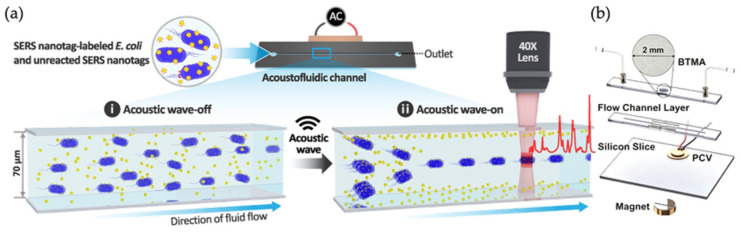
(**a**) Principle of Separating Tagged *E. coli* and SERS Nanotags Using an Acoustofluidic Channel. Reprinted with permission from ref. [78], Copyright 2026. (**b**) The structure of each part of the BTMA-SERS microfluidic chip, in which the 2 mm BTMA is embedded in the top cover of the flow channel. Reprinted with permission from ref. [19], Copyright 2026.

To further improve detection sensitivity and speed, advanced microfluidic architectures have switched from static capture to dynamic manipulation, concentrating targets in high-density zones to maximize Raman response. Park and Choo developed a lysis-free acoustofluidic SERS platform to bypass time-consuming washing and reproducibility issues [78]. Using a piezoelectric transducer (Figure 9a) to generate standing waves, the system focuses labeled *E. coli* at the central node while pushing unbound nanotags to channel walls via acoustic streaming. This wash-free strategy achieves an LOD of 1.75 × 10^5^ CFU/mL within an hour with high reproducibility. However, thermal background noise currently limits the LOD, requiring transducer optimization for early-stage sepsis diagnosis.

Addressing the trade-off between signal stability and sensitivity in single-focus systems, Dong and Wang’s group developed a SERS microfluidic chip with a barium titanate microsphere array (BTMA) [19]. Leveraging the photonic nano-jet (PNJ) effect to create a multi-focal array, the platform ensures uniform bacterial gathering via coordinated magnetic and ultrasonic forces (Figure 9b). This achieved a 5 cells/mL LOD for *P. aeruginosa* in whole blood with low RSD (~4.84%), resolving fluctuations from uneven distribution or laser misalignment. Despite superior performance, the vacuum self-assembly hot-pressing manufacturing process poses potential scalability challenges for clinical mass production.

#### 4.2.3. Spatiotemporal Metabolic Profiling in Confined Space

**Figure 10:**
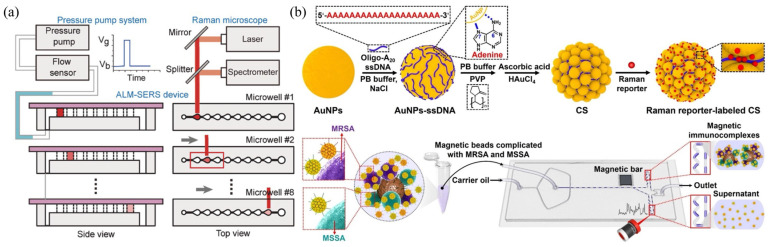
(**a**) Schematic of the ALM-SERS system. Reprinted with permission from ref. [79], Copyright 2026. (**b**) Schematic illustration of the synthetic process of CS and the detection workflow in a SERS-based microdroplet chip for mixtures of MRSA and MSSA. Reprinted with permission from ref. [80], Copyright 2026.

Microfluidic droplets—isolated, nanoliter-sized reactors that provide a confined environment for spatiotemporal studies while minimizing cross-contamination—have been employed to further explore the intricate chemical signatures of bacterial secretions. To address the challenge of competing molecular adsorption, in which high-affinity metabolites obscure others, Huang’s team developed an air–liquid microfluidics-integrated SERS system (ALM-SERS) [79]. This system uses “sequential molecular adsorption” to transport droplets across eight microwells (Figure 10a), depositing purines on the basis of their affinity. This spatiotemporal separation generates a “molecular fingerprint map,” enabling a support vector machine (SVM) to discriminate between bacterial strains of the same species but with different antibiotic resistance profiles with 100% accuracy. Although ALM-SERS resolves complex mixtures, its reliance on precise pneumatic control adds operational complexity, necessitating further automation for widespread clinical use.

To enhance reproducibility, Figure 10b presents a microdroplet sensor capable of distinguishing methicillin-resistant *Staphylococcus aureus* (MRSA) from methicillin-sensitive *S. aureus* (MSSA) [80]. The system employs a T-junction for high-throughput droplet generation, and its key innovation lies in ensemble averaging over 100 droplets. This approach reduces bulk hot-spot fluctuations and improves reproducibility six-fold (relative standard deviation ≈ 2.73%). By using PBP2a aptamers immobilized on core–satellite Au nanoparticles, the platform achieved an LOD of 2.2 × 10^4^ CFU/mL in clinical blood samples. Despite this performance, multi-step centrifugation is still required to prevent clogging, underscoring on-chip filtration as the essential next step toward a fully autonomous “sample-to-answer” device.

### 4.3. Synergistic Advantages and Translation Obstacles

The integration of SERS with microfluidics represents a transformative advancement in bacterial diagnostics, creating a synergistic environment for automated mixing, trapping, and detection within a continuous workflow. This compartmentalization reduces assay time and sample volume while enhancing sensing performance under controlled conditions, demonstrating significant potential for POCT [81,82].

A major obstacle in translating SERS-microfluidic systems from buffer-based assays to clinical diagnostics is the matrix effect of real biological samples. Blood, urine, sputum, and saliva contain proteins, salts, host cells, extracellular polymers, metabolites, and non-target microorganisms, which can compete for adsorption sites, alter nanoparticle aggregation, mask bacterial Raman fingerprints, or increase background signals. Therefore, the robustness of a SERS platform in clinical matrices should not be evaluated solely by LOD, but also by signal reproducibility, matrix tolerance, and sample-to-sample variability.

Different pretreatment and enrichment strategies address matrix effects in different ways. Magnetic enrichment can improve target concentration and separate bacteria from interfering components, while microfluidic filtration or electrokinetic trapping can reduce host cells, debris, and background molecules before Raman acquisition. Surface chemistries such as antibodies, aptamers, antibiotics, and boronic acid derivatives further improve target capture, but their specificity and stability vary across sample types. Thus, matrix-effect mitigation is highly context-dependent: strategies effective in urine or saliva may not directly translate to blood or sputum. Future SERS platforms should therefore report performance in clinically relevant matrices and compare their results with buffer-based assays to clarify their true diagnostic robustness.

Nevertheless, clinical translation still faces a “chip-simple, system-complex” hurdle. The reliance on bulky peripheral equipment, such as syringe pumps, limits portability in resource-limited settings. Moreover, continuous-flow systems often face sensitivity and signal-consistency issues due to memory effects, Taylor dispersion, and surface adsorption [83], whereas droplet-based platforms introduce additional operational complexity that requires precise pneumatic regulation. Future research should therefore focus on simplifying driving mechanisms, integrating sample pretreatment, and validating performance in raw clinical specimens.

## 5. Lateral Flow Assay-Based Bacterial Detection

### 5.1. SERS-LFA: Advancing Robust POCT

As a mature and user-friendly paper-based analytical tool, the lateral flow assays (LFAs) enable rapid quantification within complex matrices in under 30 min. This characteristic makes it a premier platform for point-of-care (POC) diagnosis of blood, urine, and other bodily fluids [84,85,86]. However, traditional colorimetric LFAs often lack sensitivity for early infections where low pathogen levels yield no “naked-eye” color change [87,88,89]. By offering notable signal augmentation, Surface-Enhanced Raman Scattering Lateral Flow Assay (SERS-LFA) overcomes this crucial bottleneck and enables accurate detection using Raman spectroscopy even in cases when the test line is colorless [90,91]. Supported by portable Raman readers [92], it is widely used to detect viruses [93], bacteria [94], and genetic markers [95]. Although lab-on-a-chip and microfluidic technologies have greatly advanced precision medicine and high-throughput biomolecular analysis [96], SERS-LFA offers a more equipment-light, user-friendly, and field-deployable format for clinical bacterial point-of-care testing (POCT), representing a practical evolution toward high-sensitivity decentralized diagnosis.

### 5.2. Structure and Mechanism

**Figure 11:**
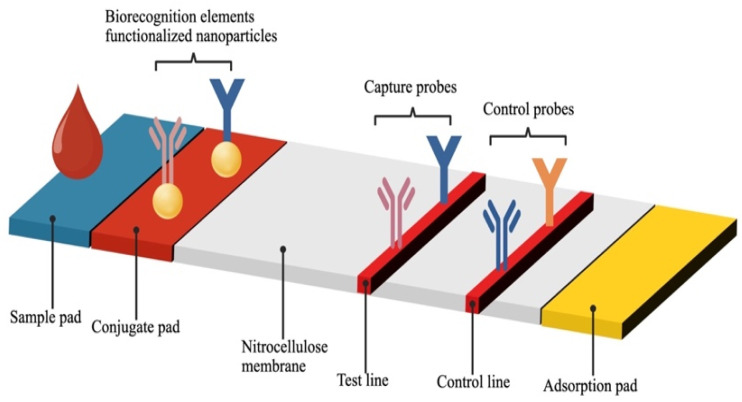
The formats and principle of lateral flow test strip.

The sensing mechanism of SERS-based lateral flow assays (SERS-LFAs) usually adopts a dual-mode signal transduction strategy that combines the visual convenience of traditional LFAs with the high-sensitivity quantification capability of Raman spectroscopy [97]. In this platform, SERS nanotags—typically gold or silver nanoparticles functionalized with Raman reporter molecules and target-specific recognition elements (e.g., antibodies or aptamers)—serve as optical probes. The physical accumulation of these metallic nanoparticles on the test line enables colorimetric detection: owing to their localized surface plasmon resonance (LSPR) properties, the dense aggregation of nanoparticles produces a visible band (e.g., red for gold nanoparticles) that allows for rapid qualitative screening [98]. Concurrently, under laser excitation, the closely packed nanoparticles generate intense electromagnetic “hot spots,” which dramatically amplify the vibrational fingerprints of the reporter molecules [99]. This amplification enables ultrasensitive quantification using portable Raman scanners, even when the nanoparticle accumulation is invisible to the naked eye. Figure 11 illustrates the typical structure of an LFA strip, highlighting the sample pad, conjugate pad, nitrocellulose membrane (with test and control lines), and absorbent pad.

The architectural design of the SERS nanotags and their interaction with target pathogens have a fundamental impact on SERS-LFA’s sensitivity and multiplexing capabilities. Recent advances have moved from simple spherical particles to sophisticated three-dimensional (3D) heterostructures that maximize hotspot density and capture efficiency.

#### 5.2.1. Structural Engineering for Enhanced Mobility and Encoding

**Figure 12:**
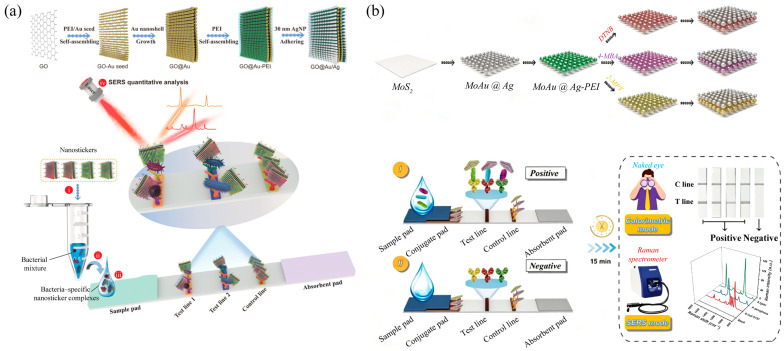
(**a**) Schematic diagram of synthesis of 3D GO@Au/Ag SERS nanosticker. Reprinted with permission from ref. [100], Copyright 2026. (**b**) Schematic of the fabrication of Raman molecule-embedded membrane-like MoDAu@Ag nanocomposites. Reprinted with permission from ref. [101], Copyright 2026.

The initial phase of innovation focuses on the physical architecture of SERS tags to overcome the size mismatch between pathogens and membrane pores (Figure 12). Wang et al. pioneered the “nanosticker” concept, employing flexible Ag satellites grafted onto graphene oxide (GO) nanosheets [100]. This hybrid 2D/3D morphology enables the tag to “wrap” around large bacteria, thereby improving the flow behavior and adhesion of the immunocomplexes, achieving a limit of detection (LOD) of 9 cells/mL. Shen et al. refined the assembly process using layer-by-layer (LbL) self-assembly on MoS_2_ nanosheets [101]. By precisely regulating the internal nanogaps via polyethyleneimine (PEI) mediation, they achieved not only superior signal reproducibility but also a sophisticated SERS-encoding strategy. This method enables the simultaneous detection of three distinct pathogens on a single test line within 16 min.

#### 5.2.2. Multimodal “Trinity” Nanozymes for Cross-Verification

**Figure 13:**
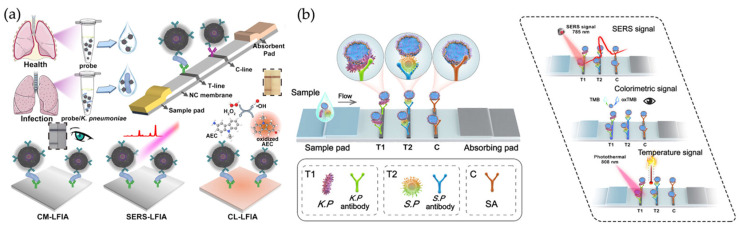
(**a**) Schematic illustration of the fabrication process for the SERS probe and the operational process of multi-model CM/CL/SERS-LFIA. Reprinted with permission from ref. [102], Copyright 2026. (**b**) Principle diagrams of the synthesis procedure of CTAC-regulated CuS-Au heterostructure and the SERS reporter and biotin-ConA modification as signal nanotags, the CuS-Au nanotags assembled LFA for the SERS/colorimetric/photothermal multimode visual/quantitative detection of S. pneumoniae and K. pneumoniae. Reprinted with permission from ref. [103], Copyright 2026.

Beyond structural optimization, the integration of peroxidase-like catalytic activity into SERS platforms provides a diagnostic “double insurance” strategy (Figure 13). To overcome the “shielding effect”, in which a catalytic shell (e.g., platinum) inadvertently attenuates the SERS signal, Zhi et al. engineered a multimetallic intra-nanogap nanozyme (Au@Au@Ag/Pt) [102]. Their triple-readout platform (colorimetric-catalytic-SERS) embeds Raman reporters within protected internal nanogaps, thereby increasing sensitivity by 200-fold compared to conventional lateral flow assays (LFAs). Similarly, Lin et al. constructed site-selective Au nanostars grown on copper sulfide (CuS) nanodiscs [103]. This spatially separated architecture promotes the generation of hot electrons under light irradiation, creating a “trinity” response that integrates SERS detection with photothermal therapy. As a result, the platform serves as a powerful tool for the rapid identification of both Streptococcus pneumoniae and Klebsiella pneumoniae in complex saliva samples, eliminating the need for time-consuming pretreatment steps. Of note, the photothermal therapy in this work is activated by a laser wavelength distinct from the excitation wavelength employed for SERS detection. In most cases, SERS substrates are intentionally designed to minimize photothermal effects at the detection wavelength. Consequently, the laser power and exposure dose applied during routine SERS measurements are unlikely to compromise bacterial viability, cell-wall integrity, or the Raman spectral profiles.

#### 5.2.3. Recognition-Element Engineering for Clinical Robustness

**Figure 14:**
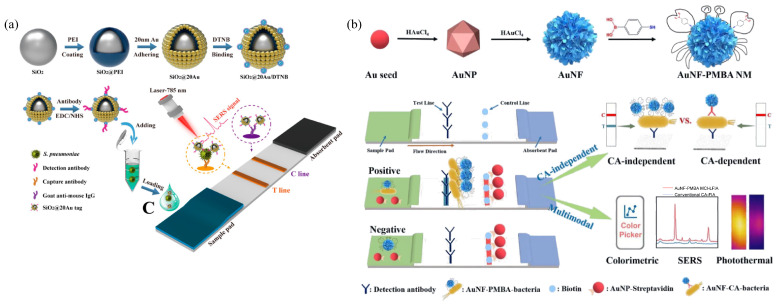
(**a**) Preparation of SiO2@Au SERS tags and SERS-ICA principle for sensitive detection of Streptococcus pneumoniae. Reprinted with permission from ref. [104], Copyright 2026. (**b**) Preparation of Au NF−PMBA NMs and principle of MCI-LFIA for bacterial detection. Reprinted with permission from ref. [105], Copyright 2026.

The third phase of evolution focuses on the recognition elements themselves, addressing the high cost and instability of antibodies in point-of-care applications. Chemical affinity-based capture using boronic acid derivatives, such as 4-mercaptophenylboronic acid (4-MPBA), has emerged as a universal alternative. However, because 4-MPBA broadly recognizes cis-diol-containing structures, it is not inherently species-specific. In polymicrobial or debris-rich clinical samples, non-target bacteria, glycoproteins, polysaccharides, or cell-wall fragments may also cause nonspecific capture or background signals. Therefore, 4-MPBA-based assays are more suitable for universal bacterial enrichment or preliminary screening, and should be combined with secondary recognition elements, magnetic washing, or species-specific spectral fingerprint analysis when precise pathogen identification is required. As shown in Figure 14a, Shen et al. developed a SiO_2_@Au SERS-tag-based immunochromatographic assay for sensitive detection of *S. pneumoniae*, achieving an LOD of 46 cells/mL and demonstrating respiratory-pathogen-oriented strip detection with higher sensitivity than conventional colorimetric ICA [104]. To further boost sensitivity, Wu et al. developed Au nanoflower-PMBA “nanocrabs” with high-density hotspots from sharp branching (Figure 14b) [105]. Coupled with switchable detection modes (visual, Raman, photothermal), this enables high-precision *E. coli* identification in clinical urine within 45 min without expensive biological ligands. Similarly, the 4-MPBA-based theranostic platform developed by Gao et al. (Figure 7a) successfully leveraged this chemical affinity to capture bacteria in whole blood, while utilizing Prussian Blue for subsequent photothermal pathogen elimination [62].

### 5.3. Clinical Feasibility and Community Relevance

The translation of SERS-LFA from laboratory research to clinical application depends not only on analytical sensitivity but, more importantly, on operational robustness and socio-economic accessibility. At the community healthcare level, feasibility is determined by diagnostic accuracy, reproducibility, ease of use, and cost-effectiveness rather than by the LOD alone.

SERS-LFA offers a decentralized alternative to traditional centralized microbiology, which typically requires specialized personnel and multi-day incubation. However, challenges remain regarding the environmental stability of SERS tags, batch-to-batch reproducibility, and robustness in complex clinical samples. Although immobilization on the paper matrix successfully stabilizes plasmonic tags, the membrane lateral flow non-uniformity and the paper matrix-driven variance remain non-negligible. Furthermore, although paper substrates are inexpensive, the cost of portable Raman readers may still pose an economic barrier for small clinics or resource-limited settings. Portable Raman readers also generally have lower spectral resolution, signal-to-noise ratio, and wavelength stability than benchtop instruments, which may obscure weak fingerprint peaks and affect strain-level discrimination or multiplexed detection.

For the system to be truly relevant at the community level, its design must accommodate operation by non-experts and tolerate minor variations among real specimens, such as urine or saliva samples from different patients. From a broader commercialization perspective, SERS-based bacterial diagnostics are still at an early translational stage. Most bacteria-oriented SERS assays remain at the proof-of-concept or small-scale clinical-sample validation stage, while large-scale multicenter clinical trials and regulatory-approved SERS diagnostic kits remain limited. Therefore, future development should shift from simply breaking LOD records to ensuring manufacturable substrates, stable shelf life, automated pretreatment, affordable and calibrated portable Raman readers, standardized spectral databases, and external validation with clinically relevant samples.

### 5.4. Comparison of Integration Strategies: Depth vs. Breadth

To systematically evaluate the technological landscape, a comparative analysis across different types of SERS platforms is presented. This comparison is not intended to determine a winner but rather to establish a diagnostic hierarchy based on clinical needs (Table 3).

The distinction between microfluidic SERS and SERS-LFA exemplifies a classic trade-off between controllability and simplicity. Microfluidics creates an active environment in which fluid dynamics can be precisely tuned to optimize the formation of electromagnetic hot spots. For clinical scenarios such as sepsis, where every minute and every colony-forming unit (CFU) matters for patient survival, this enhanced controllability translates into greater accuracy and reliability.

In contrast, SERS-LFA operates on a passive principle. Its elegance arises from its reliance on capillary forces, thereby eliminating failure points associated with mechanical pumps. Going forward, SERS diagnostics will likely evolve along two divergent tracks. It would be unreasonable to expect LFA to fully replace the high-throughput capabilities of microfluidics, or for microfluidics to match the portability of LFA. Instead, a successful clinical ecosystem will employ LFA for rapid triage at the point of care, while reserving microfluidic SERS platforms for in-depth antibiotic susceptibility testing (AST) or the analysis of complex co-infections. This hybrid approach maximizes the clinical utility of SERS technology across the entire healthcare spectrum.

To propose a standardized framework beyond LOD, we suggest evaluating SERS platforms using six key performance indicators: (i) LOD and specificity, (ii) total assay time, (iii) cost per test, (iv) operational complexity, (v) matrix tolerance (recovery rate in clinical matrices), and (vi) batch-to-batch reproducibility. These criteria should be weighted according to the diagnostic scenario—POC prioritizes speed and simplicity, while reference labs emphasize sensitivity and reproducibility. Such a framework facilitates balanced, clinically meaningful comparisons.

## 6. Conclusions and Future Perspectives

In summary, the landscape of SERS-based bacterial detection has shifted from fundamental optical labels toward sophisticated, process-oriented nanoplatforms. This review has highlighted a purposeful movement in the field toward addressing the actual impediments in the diagnostic workflow, in contrast to earlier studies that primarily focused on maximizing electromagnetic enhancement through colloidal geometry. The transition from free-floating colloids to engineered solid substrates, and further toward the current synergy achieved with microfluidics and LFAs, reflects a hierarchy of clinical needs. While colloids offer superior three-dimensional sample interaction, solid interfaces enhance reproducibility in biological electrolytes. Ultimately, integrating these materials into microfluidic chips or LFA strips bridges the gap between benchtop innovation and bedside utility.

However, widespread clinical adoption faces challenges that extend beyond analytical sensitivity. Complex clinical matrices—blood, sputum, saliva—often mask SERS fingerprints through competitive adsorption. Beyond matrix effects, there exists a critical “operational robustness gap.” A platform that cannot tolerate minor variations in sample viscosity or operator error may perform flawlessly in a controlled laboratory setting but fail in a busy clinical environment. Furthermore, the “chip-simple, system-complex” dilemma continues to hinder portability, as the benefits of a miniaturized sensing interface are often negated by the high cost and bulkiness of Raman spectrometers. To overcome these obstacles, the field must redirect its focus from pursuing record-breaking limits of detection toward ensuring the long-term stability, cost-effectiveness, and batch-to-batch reliability of the complete diagnostic system.

The future of SERS-based bacterial detection applications lies in a seamless transition to real-world applications, emphasizing direct pathogen identification without intensive cultivation [26]. Integrating microdevices with portable Raman spectrophotometers is essential for rapid on-site diagnostics [106,107]. Moreover, although algorithmic correction cannot fully replace the need for robust hardware and standardized acquisition parameters, embedding machine learning (ML) and deep learning (DL) into system software will automate data processing and species recognition [108,109,110]. Convolutional neural network (CNN) models have already achieved 99.7% accuracy for pathogen identification within 15 min [111], offering additional insights such as early prognosis [15,112]. Bridging the laboratory-to-clinic gap requires multidisciplinary collaboration [113] to build large algorithmic databases and ensure operational efficacy in real-world clinical settings [114,115]. Nevertheless, the clinical use of ML-assisted SERS still requires more than high classification accuracy. Conventional ML methods such as PCA/LDA, SVM, and random forests can provide relatively interpretable classification based on selected spectral features, whereas deep learning models such as CNNs are powerful for extracting high-dimensional spectral patterns but may suffer from limited interpretability and dataset dependence [116]. Therefore, future ML-assisted SERS diagnostics should emphasize interpretable model design, standardized spectral preprocessing, shared and well-annotated datasets, calibration transfer, and external validation across instruments, laboratories, and clinical matrices [117,118,119].

## Figures and Tables

**Table 1:** Topical scope, organizational logic, and discussion focus.

Review	Topic Scope	Organizational Logic	Discussion Focus
Wang et al., 2021 [25]	SERS test strips	LFA/VFA format, SERS labels, applications	Strip design POCT applications
Usman et al., 2023 [26]	Bacterial pathogen identification	SERS principles, label-free/labeled detection, substrates, chemometrics	Pathogen identification strategies
Tang et al., 2024 [28]	Label-free SERS for pathogenic microbial identification	Raman/SERS fundamentals, label-free detection, computational analysis	Label-free spectra Machine learning
Cialla-May et al., 2024 [29]	Biomedical SERS	Sample pretreatment, substrates, detection strategies, biomedical applications	Broad biomedical SERS applications
This review	SERS-based bacterial	Colloidal probes, solid substrates, microfluidic chips, SERS-LFA	Platform evolution toward clinical diagnosis

**Table 2:** Comparison of diagnostic performances of different SERS in bioassays.

Platform	Materials	Tag	Target	LOD	Assay Speed	Cost Effectiveness	Sample	Ref.
Colloidal	Rau MNPs-WGA	DTNB	*E. coli*	8 cells/mL	30–60 min	Medium	Human urine	[36]
Colloidal	Fe_3_O_4_@Au@ 4−MBA	DTNB, 4-MBA	*S. aureus*	1 cell/mL	30–60 min	Medium	Human urine	[17]
Colloidal	ConA-Fe_3_O_4_@SiO_2_ NPs	4-NTP	*S. aureus*	24 CFU/mL	30–60 min	Medium	Human serum	[41]
Colloidal	Fe_2_O_4_@Au-Ab	4-MBA	*S. aureus*	7 CFU/mL	>60 min	Medium	Mouse blood	[43]
Colloidal	BP@MoS_2_	N/A	*E. coli*	1.02 × 10^4^ CFU/mL	<15 min	Low	PBS, 0.9% NaCl	[45]
Colloidal	MB-Au NPs	Cy5	*E. coli*	5.9 × 10^3^ CFU/mL	>60 min	Medium	Human urine	[44]
Colloidal	PolyA-DNA Au MNPs	R6G	*S. ty*	4 CFU/mL	15–30 min	Medium	Chicken, milk	[66]
Colloidal	M13 bacteriophage	DTTC	*E. coli*	0.5 CFU/mL	30–60 min	Medium	Mouse blood	[37]
Colloidal	Fe_3_O_4_@SiO_2_@Ag	DTNB, MPBA	*S. aureus*, *E. coli*	1 CFU/mL	30–60 min	High	Milk	[67]
Colloidal	Fe_3_O_4_@SiO_2_–Au	DTNB	*E. coli*	10 CFU/mL	30–60 min	High	N/A	[68]
Substrate	Ag-Si NWs	N/A	12 types	100 CFU/mL	<15 min	High	Synthetic urine	[56]
Substrate	pAu/G/PBA	PB, 4-MB	*S. ty*; *S. aureus*	10 CFU/mL	30–60 min	High	Mouse blood	[68]
Substrate	SAM/pAu	4-MBN	*S. aureus*	10 CFU/mL	30–60 min	High	Human blood	[62]
Substrate	cDNA-Au MPs	Cy5	*E. faecium*	0.035 nM	30–60 min	Medium	Human Blood	[63]
Substrate	Ag NPs@PDMS sponge	N/A	6 types	1 CFU/mL	<15 min	Low	Milk	[65]
Substrate	PDMS-NP-Ag	N/A	*E. coli*	10^4^ CFU/mL	<15 min	Low	N/A	[69]
Substrate	Au@Ag/Cu-MIM	4-MPBA	*E. coli/S. aureus*	10 CFU/mL	30–60 min	High	Human blood	[70]

**Table 3:** Consolidated platform-level comparison of SERS-based bacterial detection strategies.

Platform	LOD/Time	Strength	Limitation	Clinical Usability	Best-Fit Scenario
Colloidal probes	~1–10^3^ CFU/mL; minutes to hours	High target-probe collision efficiency; easy surface functionalization	Aggregation, batch variation, matrix instability	Moderate; often requires controlled sample preparation	Rapid enrichment and sensitive detection in liquid samples
Solid substrates	~1–10^4^ CFU/mL; minutes to hours	Improved signal stability and substrate reproducibility	Limited bacteria- substrate contact; large-area hotspot uniformity remains difficult	Moderate; easier to standardize than colloids	Reproducible spectral acquisition and chip-based sensing
Microfluidic SERS	~1–10^5^ CFU/mL; less than 1 h	Integrates pretreatment, enrichment, mixing, and detection	Pumps/tubing, channel clogging, system complexity	High analytical control but weaker field portability	Complex samples and sample-to- answer workflows
SERS-LFA	~1–10^3^ CFU/mL; less than 1 h	Low cost, simple operation, POCT-compatible	Lower flow controllability; limited multiplexing capability due to test-line design	High; suitable for non-expert operation	Frontline screening and decentralized diagnosis ^1^

^1^ Typical performance ranges are summarized from representative studies listed in Table 2 and may vary with target bacteria, sample matrix, and assay design.

## Data Availability

No new data were created or analyzed in this study. Data sharing is not applicable to this article.
